# Supplementary material for: Evolution shapes interaction patterns for epistasis and specific protein binding in a two-component signaling system
Source: Commun Chem. 2024 Jan 17;7:13. doi: 10.1038/s42004-024-01098-2 (PMC10794238; doi:10.1038/s42004-024-01098-2)
Supplement: Supplementary file 2 — Supplementary Materials [file 42004_2024_1098_MOESM2_ESM.pdf]

Supplementary Information for

Evolution shapes interaction patterns for epistasis and specific protein binding in a two-component signaling system

Zhiqiang Yan and Jin Wang

Jin Wang.
E-mail: jin.wang.1@stonybrook.edu

This PDF file includes:

Supplementary text
Figs. S1 to S10
Tables S1 to S9
Legends for Dataset S1 to S5
SI References

Other supplementary materials for this manuscript include the following:

Datasets S1 to S5

Supporting Information Text

Supplementary Methods

Physical principles of protein binding and evolution. Protein binding here refers to how two protein partners interact with each other and form functional complex structure. Protein evolution refers to how protein's sequence and structure change through mutation and selection. To understand physical principles of protein binding and evolution, one can assume that there are two interacting proteins, their sequences have been evolved to be able to fold and bind independently. The total energy of a binding complex conformation thus can be expressed as

$$E = E_1 + E_2 + E_{12}, \tag{1}$$

where E_1 and E_2 are the energies of the conformations of protein 1 and protein 2, and E_{12} is the energy between them. The total energy is assumed to be the sum of independent interactions between residues. According to the central limit theorem, the probabilities of the energy states of binding complex follows Gaussian distribution, which is

$$P(E) = \frac{1}{\sqrt{2\pi\Delta E^2}} \exp[-\frac{(E-\overline{E})^2}{2\Delta E^2}], \tag{2}$$

where $\overline{E}$ is the average of total energy, which is $\overline{E} = \overline{E}_1 + \overline{E}_2 + \overline{E}_{12}$, and ΔE^2 is the energy fluctuation of total energy, which is $\Delta E^2 = \Delta E_1^2 + \Delta E_2^2 + \Delta E_{12}^2$. Assuming that the total number of binding conformations is Ω_0, the entropy of each energy state can be obtained ,

$$S(E) = K_B \ln(\Omega(E) = K_B \ln(\Omega_0 P(E)) = S - K_B \frac{(E-\overline{E})^2}{2\Delta E^2}, \tag{3}$$

where $\Omega(E)$ is the number of binding conformations at energy state E. S_0 is the total entropy $S_0 = K_B \ln \Omega_0$. Through the thermodynamic relation $\partial S/\partial E = 1/T$, the energy of the system as a function of the temperature is

$$E(T) = \overline{E} - \frac{\Delta E^2}{K_B T}, \tag{4}$$

Combining equation 3 and 4, one can obtain the entropy as a function of the temperature, which is

$$S(T) = S_0 - \frac{\Delta E^2}{2K_B T^2}. \tag{5}$$

With the thermodynamic expressions of the energy and entropy, the Helmholtz free energy of the system as a function of temperature can be expressed as

$$F(T) = \overline{E} - TS_0 - \frac{\Delta E^2}{2K_B T}. \tag{6}$$

Naturally occurring complexes normally have a native functional state at which both the entropy and energy variance or roughness are zero. Then the free energy of the native state equals E_N. By equaling the free energies of native state and non-native state, i.e.$E_N = \overline{E} - TS_0 - \frac{\Delta E^2}{2K_B T}$, one can get a characteristic temperature, that is

$$T_b = \frac{\delta E}{2S_0}(1 + \sqrt{1 - \frac{2S_0 \Delta E^2}{\delta E^2}}), \tag{7}$$

T_b can be considered as a binding transition temperature between native and non-native binding state. For an infinite high temperature, the entropy $S(T)$ is approximately S according to the equation 5. As the temperature is lowered the entropy decreases until another characteristic temperature is reached, that is

$$T_g = \sqrt{\frac{\Delta E^2}{2K_B S_0}}. \tag{8}$$

At or below this temperature, the system encounters entropy crisis where the system is trapped into one of the frozen states. This characteristic temperature is called trapping temperature (1–3). To guarantee the system can reach the native state rather than trapping in the frozen state, T_b should be larger than T_g, i.e. T_b/T_g should be larger than 1. Combining equation 7 and 8, one can get

$$\frac{T_b}{T_g} = \Lambda^b + \sqrt{(\Lambda^b)^2 - 1}, \tag{9}$$

where

$$\Lambda^b = \sqrt{\frac{K_B}{2S}} \frac{\delta E}{\Delta E}. \tag{10}$$

The larger the ratio T_b/T_g is, the less chances of the system can be trapped on the way to the native state. On the other hand, Λ^b is a quantitative measure of the landscape topography of protein binding. The larger the Λ^b is, the more funneled protein binding energy landscape shape is against the vast number of states and roughness, i.e. minimal frustration principle. The

relationship between the $\frac{T_b}{T_g}$ and Λ^b indicates that maximizing $\frac{T_b}{T_g}$ is equivalent of maximizing the value of Λ^b . It provides a practical implementation of the principle of minimal frustration for protein binding. Optimization of Λ^b guarantees the kinetic accessibility of the native state (4**?** –6). At a particular temperature higher than T_g, the probability $P(E)$ of sampling any conformations with energy E is weighted by the Boltzmann factor $\exp[(E-\overline{E})/K_BT]$. Thus, the probability of the system in its unique native state can be obtained through equation 2, which is

$$P_N = \frac{\exp[-\frac{(E_N-\overline{E})}{K_BT}]}{Z}, \tag{11}$$

and the probability that the sequence in the non-native binding state is given by

$$P_D = \sum_{E>E_N} \frac{P(E)\exp[-\frac{(E-\overline{E})}{K_BT}]}{Z}, \tag{12}$$

where Z is the partition function of the canonical ensemble. Thus, the thermodynamic stability of the native ground state is quantified as

$$\Delta G^b = -K_BT\ln(\frac{P_N}{P_D}) = E_N + K_BT\ln[\sum_{E>E_N} P(E)\exp(\frac{-E}{K_BT})]. \tag{13}$$

A funnelled and minimally frustrated landscape with stable native state can be achieved if the binding complex evolves with the optimization of ΔG^b for thermodynamic stability and Λ^b for kinetic accessibility. The expressions of Λ^b and ΔG^b provide mathematical foundations and formulations which can be quantified as the selection force or fitness of the protein evolution. The principle of minimal frustration can be realized by optimization of Λ^b and ΔG^b during evolution. The expressions of Λ^b and ΔG^b here incorporates both folding and binding requirements. For independent folding and binding, the binding can be considered as rigid binding of two already folded proteins. In this case, the expressions of Λ^b and ΔG^b only represents the binding requirement, and the folding requirement can be expressed with Λ^f and ΔG^f as derived in previous paper (7).

Setup for protein binding and evolution of TCS. The complex structure of TCS contains HK and RR (Figure 1). HK is composed of two chains which constitutes one DHp domain and two CA domains, and RR is composed of two identical and separated Rec domains. To identify the interaction pattern which are specific for binding, the evolution simulations of Rec domain were carried out separately under two conditions, i.e. the presence of HK as the binding partner and the absence of HK. This kind of setup for the binding and evolution of TCS is based on two aspects. First, only homologous sequences (PF00512) of DHp domain are available in the Pfam database, this leads to the statistical analysis being based on the length of DHp domain rather than the whole HK. From the contact map between Rec domain and HK (Supplementary Fig. S7b), Rec domain not only interacts with DHp domain, but also CA domain. This means that the whole length of HK (including DHp and CA domain) should be considered in the evolution. In other words, the native sequences involve the length of DHp domain while the evolved sequences involve the length of the whole HK. The comparison between them would be incomplete. Second, due to C2 symmetry of the complex structure, the binding between HK and RR can be represented by the binding between HK and one Rec domain since two Rec domains locate at the opposite surface of HK and have no interactions with each other. Rec domain is an independent evolution and folding unit. This is why the sequence of one Rec domain was set to be evolved at the presence/absence of the whole HK. Alternatively, if the sequence of HK was set to be evolved, both two separated Rec domains should be considered simultaneously as binding templates and two binding surfaces between Rec domains and HK should be included in the structure-oriented evolution simulations. In addition, the whole HK contains two chains and three domains, which also involves both folding and binding. In this case, the evolution of HK at the presence of Rec domains would be driven by folding and binding fitness within HK, as well as binding fitness between Rec domains and HK. This complexity is beyond the reach of our current evolution methods. Thus, only the evolution of Rec domain was carried out at the condition with or without the presence of HK.

Computation of Residue-level frustration index with Customized code. Since the quantification of local frustration in proteins was proposed (8), the frustration index has been widely measured by the frustratometer algorithm (9). The algorithm evaluates energies according to the associative memory Hamiltonian water-mediated (AMW) potentials(10). There are three ways to compute local frustrations, i.e. configurational frustration, mutational frustration and residue-level frustration. In our computation, we chose residue-level frustration because it can be compared directly with the position conservation and hydrophobic preference in the analysis. For the computations, our own customized codes for residue-level frustration with C language was developed due to two aspects. First, in accordance with the residue-level potential (i.e. Miyazawa-Jernigan (MJ) matrix) used in our evolution simulations with residue-level mutation, the MJ matrix rather than AMW potentials was also employed to compute residue-level frustrations. Second, the residue-level frustration indexes were averaged over a number of native sequences (=4069) or evolved sequences (=5000) mapping onto the target structure, this requires customized code to carry out processing of multiple sequences rather than once a time on the server.

The frustratometer algorithm with AMW potentials has been widely used and cited. To validate our customized code with MJ matrix in computing local frustrations, we have compared the frustration indexes at residue level for two cases. One is the Rec domain of TCS (PDB ID: 3DGE, chain C) which we employed as the protein model, the other is an example protein

(PDBID: 4ZKQ, chain A) which was employed as one of the protein models to show the results of frustration indexes on the Frustratometer server (http://frustratometer.qb.fcen.uba.ar). The frustration index results of these two cases computed with AMW potentials on the server can be obtained from the links (http://frustratometer.qb.fcen.uba.ar/results/201637193147661102, http://bonarda.qb.fcen.uba.ar/results/202312734041952360) and the residue-level frustration indexes are shown Supplementary Dataset S5. The residue-level frustration index results computed by our customized code with MJ matrix are also shown in Supplementary Dataset S5.

It can be seen that the correlation coefficients between two versions for these two cases are 0.65 and 0.66 respectively (Supplementary Fig. S9a and b). These correlation coefficients suggest that majority of the frustration indexes from two versions are consistent (Supplementary Fig. S9c and d). By mapping the differences of frustration indexes ($\Delta F = |F_{custom} - F_{server}|$) onto the structure, we found that the positions with large differences mainly located at the structural surfaces, especially the loop regions on the surfaces (Supplementary Fig. S9e and f). In detail, if we set a threshold $\Delta F > 1.6$ (Supplementary Fig. S9c and d) for choosing the positions with large differences between two versions, for Rec domain these position are intensively located at the loop regions (position 43 to 45 and position 70 to 76). These positions have no overlaps with those which were identified as important positions for functional binding of the Rec domain (Figure 2, 3, 4 and 7). In other words, the observed results from our customized and modified version of calculating residue-level frustration index are robust and wouldn't be changed if the original version with AMW hamiltonian were employed. The differences between two versions mainly locate at the loop regions could be due to that AMW potentials contains not only pairwise contact term, but also a single-residue burial term which accounts for the solvent exposure of the residue. The loop regions on the structural surface generally have few contacts and exposed to the solvent. In this sense, AMW could more accurate to represent the surrounding context of the residue positions at loop regions. For referring and assessing our code, please see the link of github repository (https://github.com/ZQYanUCAS/EvolutionShapesInteractionPattern).

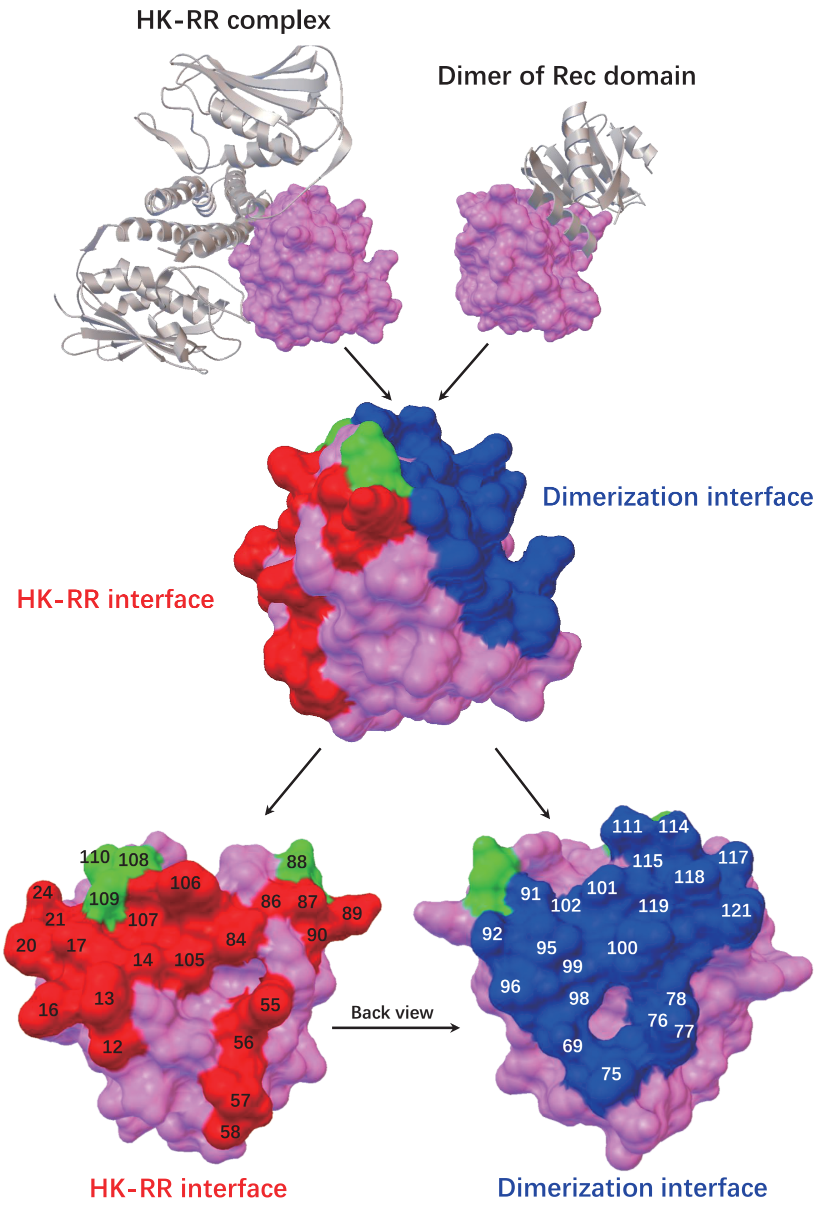

Fig. S1. Functional binding surfaces of Rec domain. The upper panel shows the structures of Rec domain binding with HK (PDB ID 3DGE), and its dimerization (PDB ID 1ZES), the Rec domain is displayed with purple molecular surface; the middle panel shows two binding interfaces: HK-RR interface and dimerization interface; the bottom panel shows positions participating in the binding surfaces. HK-RR interface is colored in red and dimerization interface is colored in blue, the positions colored in green are the overlapping positions for these two binding interfaces; the position on the binding surface is defined once it has at least one contact with any positions in the binding partner.

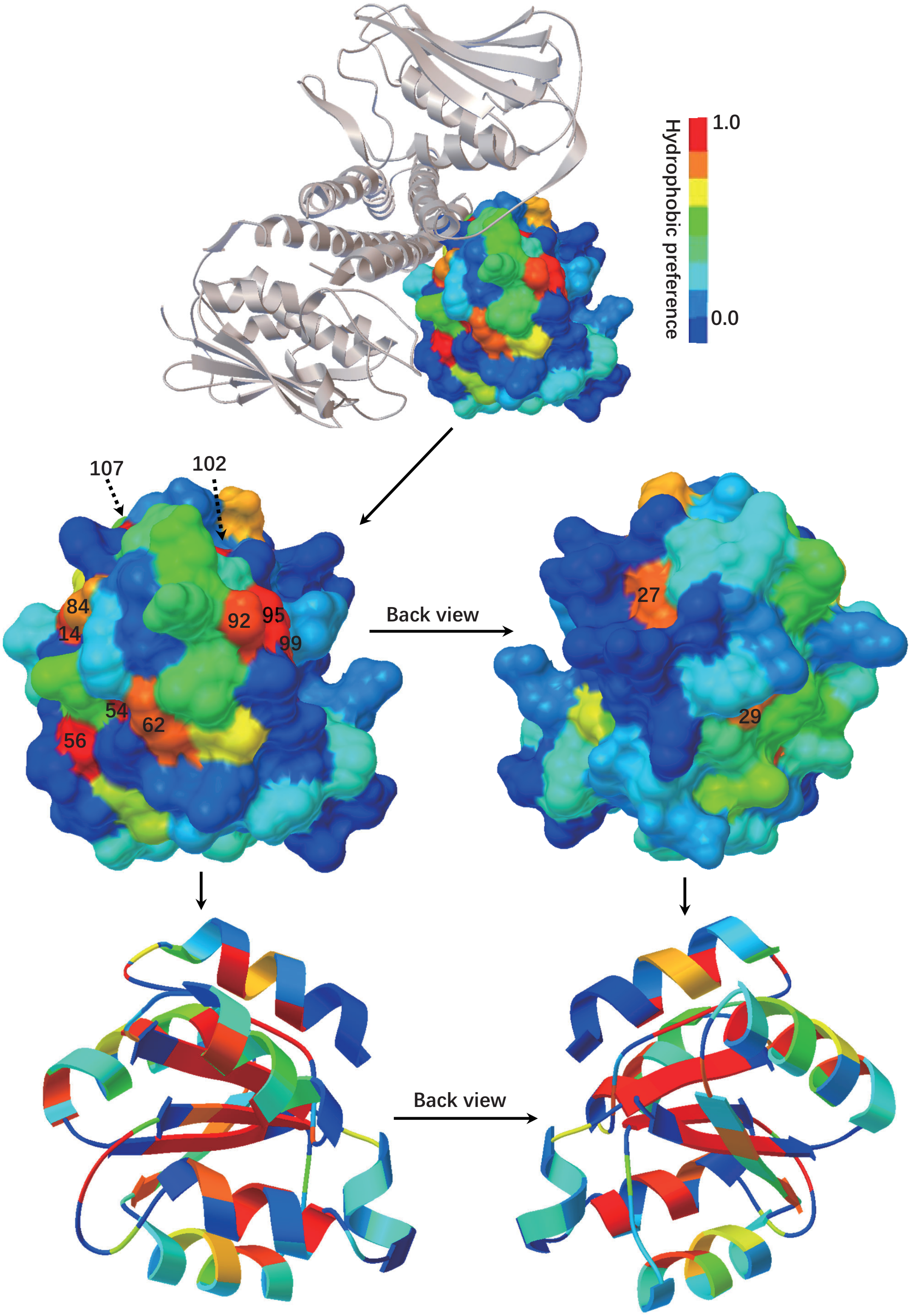

Fig. S2. Hydrophobic preferences of the positions across the Rec domain. The upper panel shows the structure of Rec domain binding with HK, the color bar represents hydrophobic preference; the middle panel labels the positions with highly hydrophobic preferences on the structural surface; the bottom panel shows the hydrophobic preferences of the positions on the ribbon structure, a hydrophobic core is obviously located inside the structure.

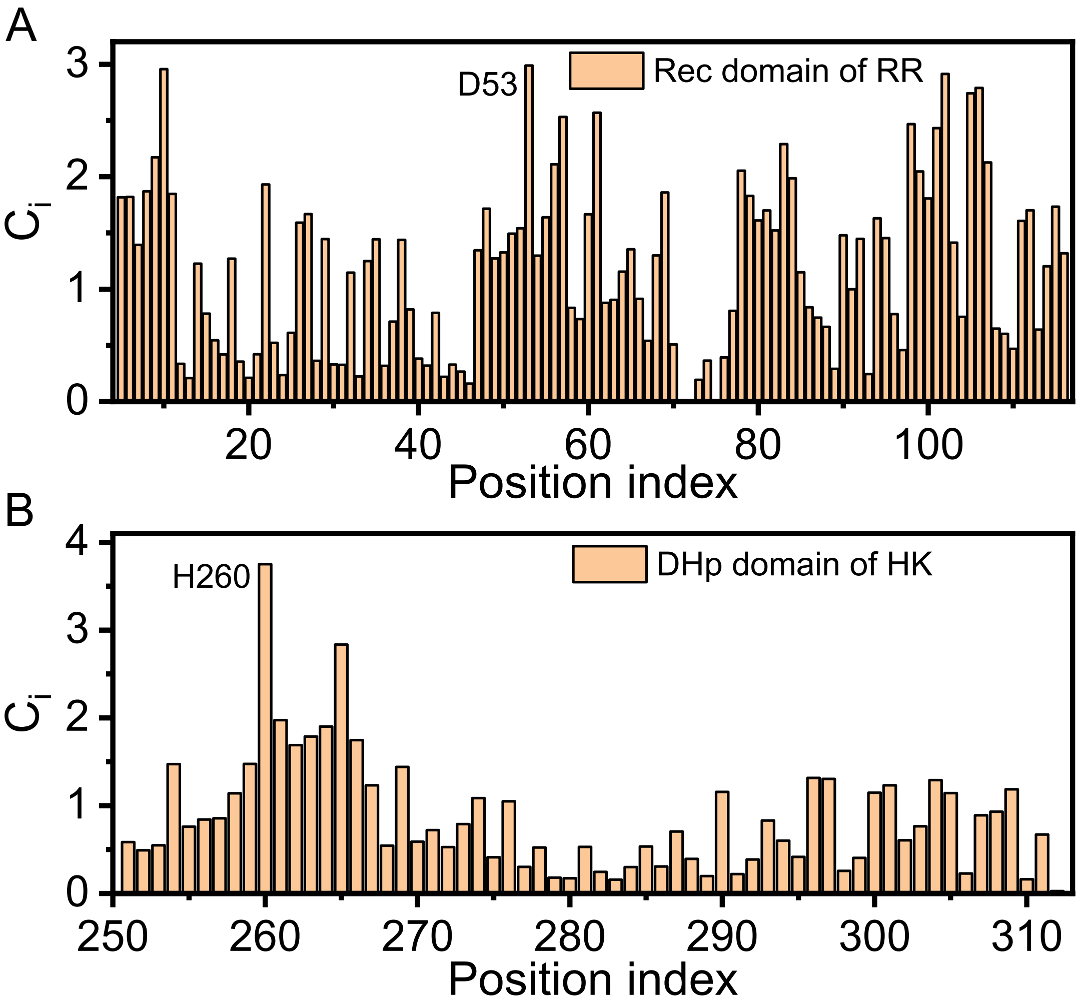

Fig. S3. Position conservation (C_i) on the sequences of Rec domain (A) and DHp domain (B). The most two conserved positions D53 and H260 responsible for auto-phosphorylation, phosphotransfer, and phosphatase activities are labeled.

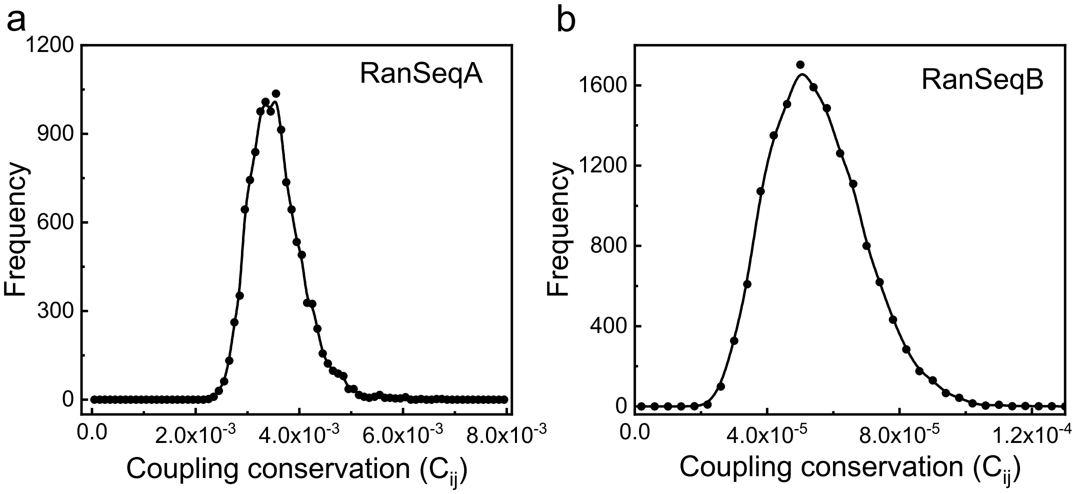

Fig. S4. Gaussian distribution of coupling conservation (C_{ij}) frequency from MSA of random sequences. (a) The random sequences (named as RanSeqA, Supplementary Dataset S3) are generated as the following. First, the lengths of the sequences were set as the same as the native sequences, that is 62 positions for DHp domain of HK, and 110 positions for Rec domain of RR. The number of random sequences were set as 5000. Second, for each position of a sequence, the amino acid type was randomly selected from 20 types without any bias, and also the amino acid type of each position is independently assigned. i.e. the amino acids were assigned without any background frequencies or any other feature of natural proteins. (b) The random sequences (named as RanSeqB, Supplementary Dataset S4) are generated as the same as RanSeqA but with background frequencies of amino acid in natural proteins (Supplementary Table S7). The frequency-magnitude relationship of the coupling conservation extracted from these two kinds of random sequences are near zero and follows Gaussian distribution. This distribution stands in contrast to the power-law distribution from the native sequences since there are no obvious highly coupling conservations and all the values approach zero.

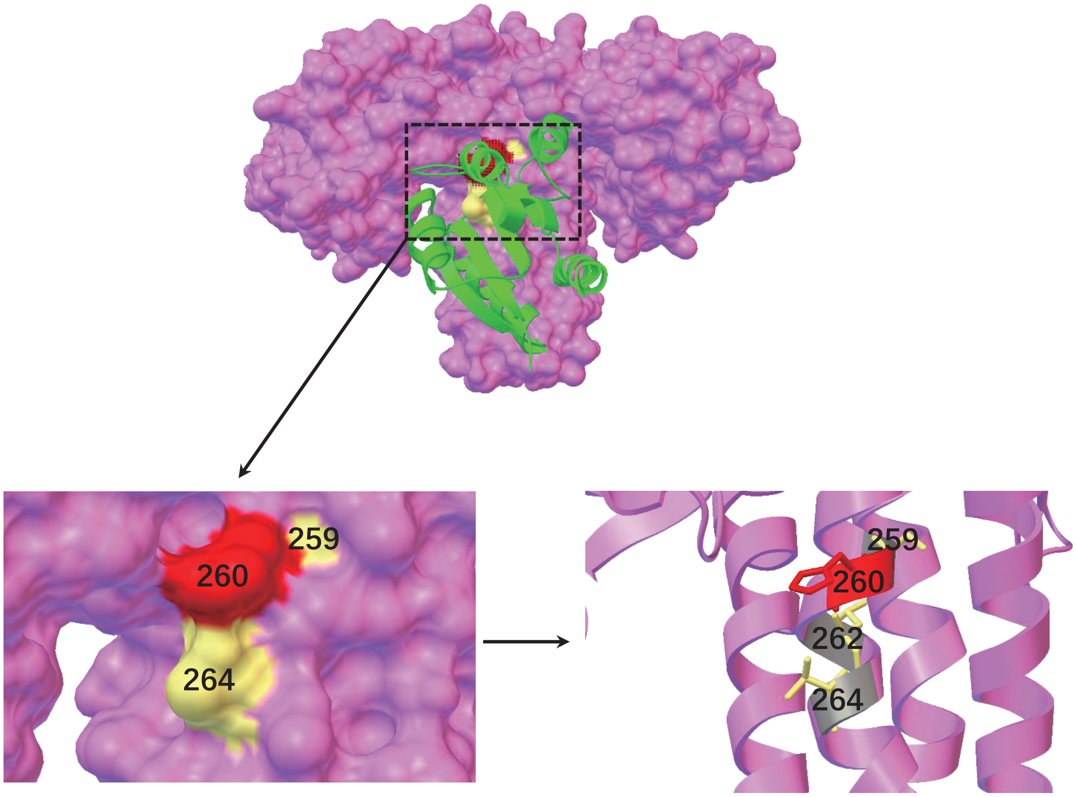

Fig. S5. Three positions in DHp domain with highly intermolecular coupling conservations. The positions (259, 262 and 264) are spatially near phosphoacceptor/phosphodonor position 260 and labeled, the structure in green is Rec domain while the structure in purple is HK.

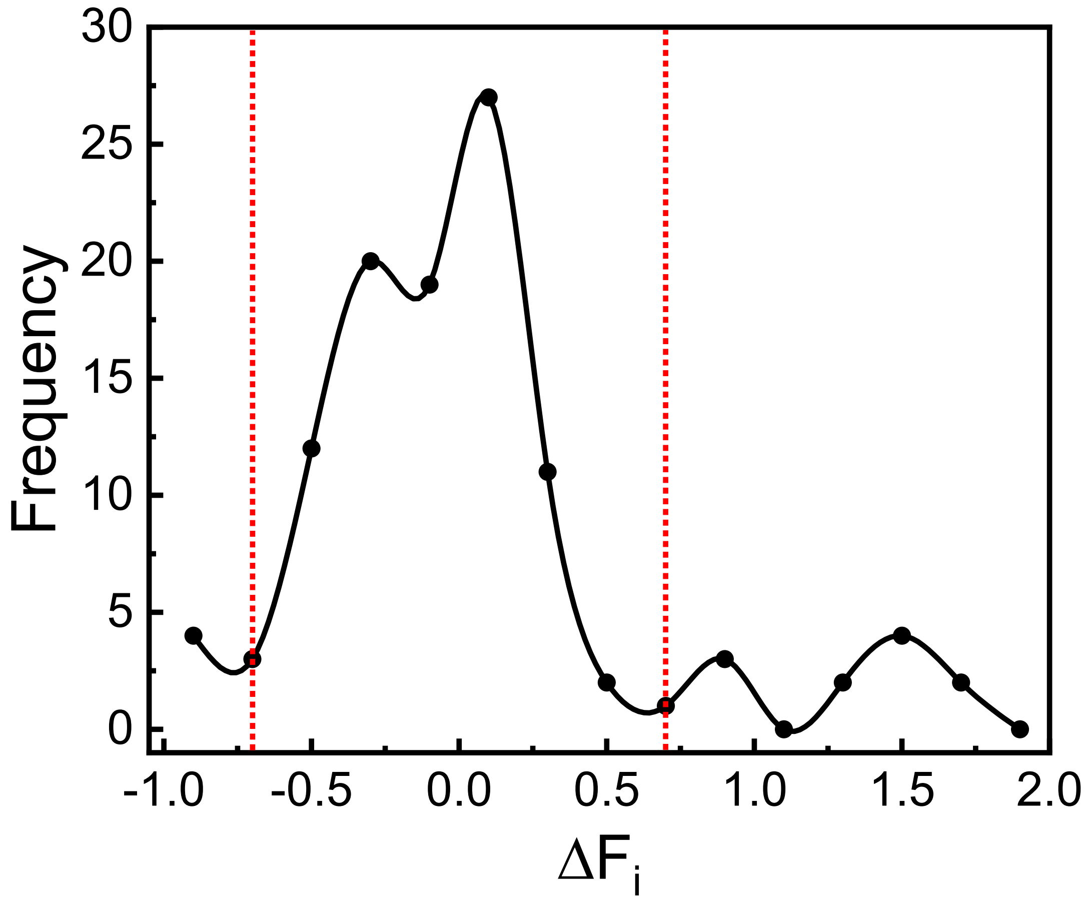

Fig. S6. Frequency distribution of $\Delta F_i = F_i^{FBS} - F_i^{FS}$. The high peaks and low peaks can be separated by $|\Delta Fi| = 0.7$ (red dotted lines), the high peak means most of the positions have small frustration changes, while those low peaks indicate some positions have large frustration changes when the binding is considered in the evolution simulation.

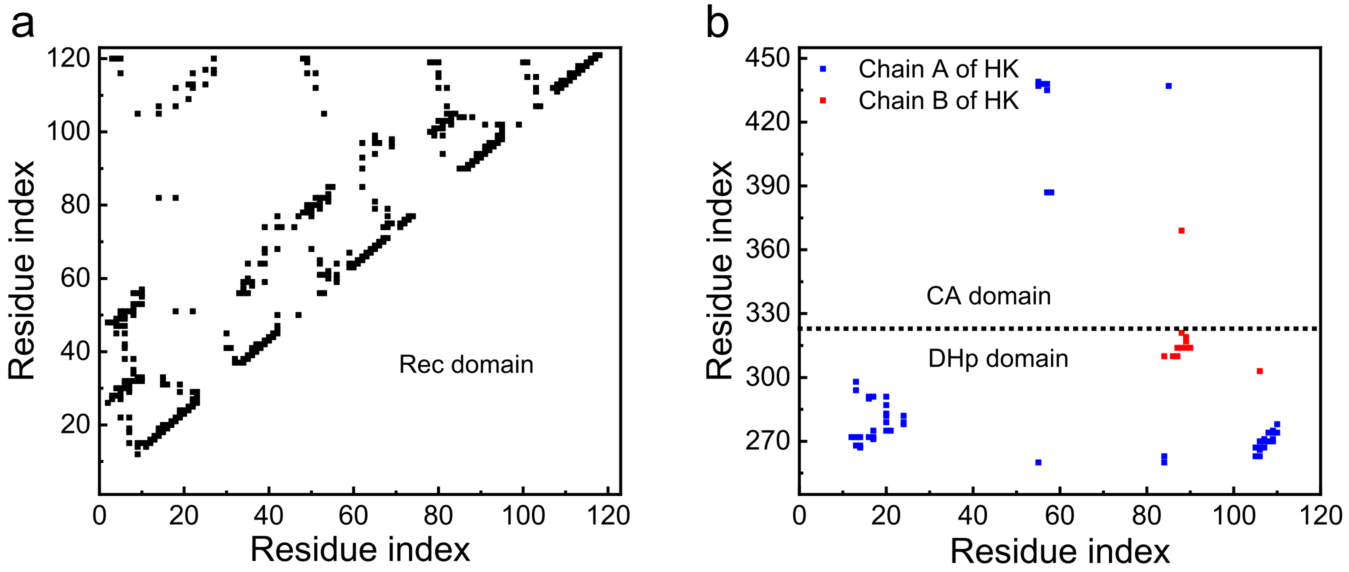

Fig. S7. Contact maps of Rec domain and its binding with HK. (a) Contact map of Rec domain. (b) Contact map of Rec domain binding with HK, the dotted line separates the contacts of Rec domain binding with DHp domain and CA domain.

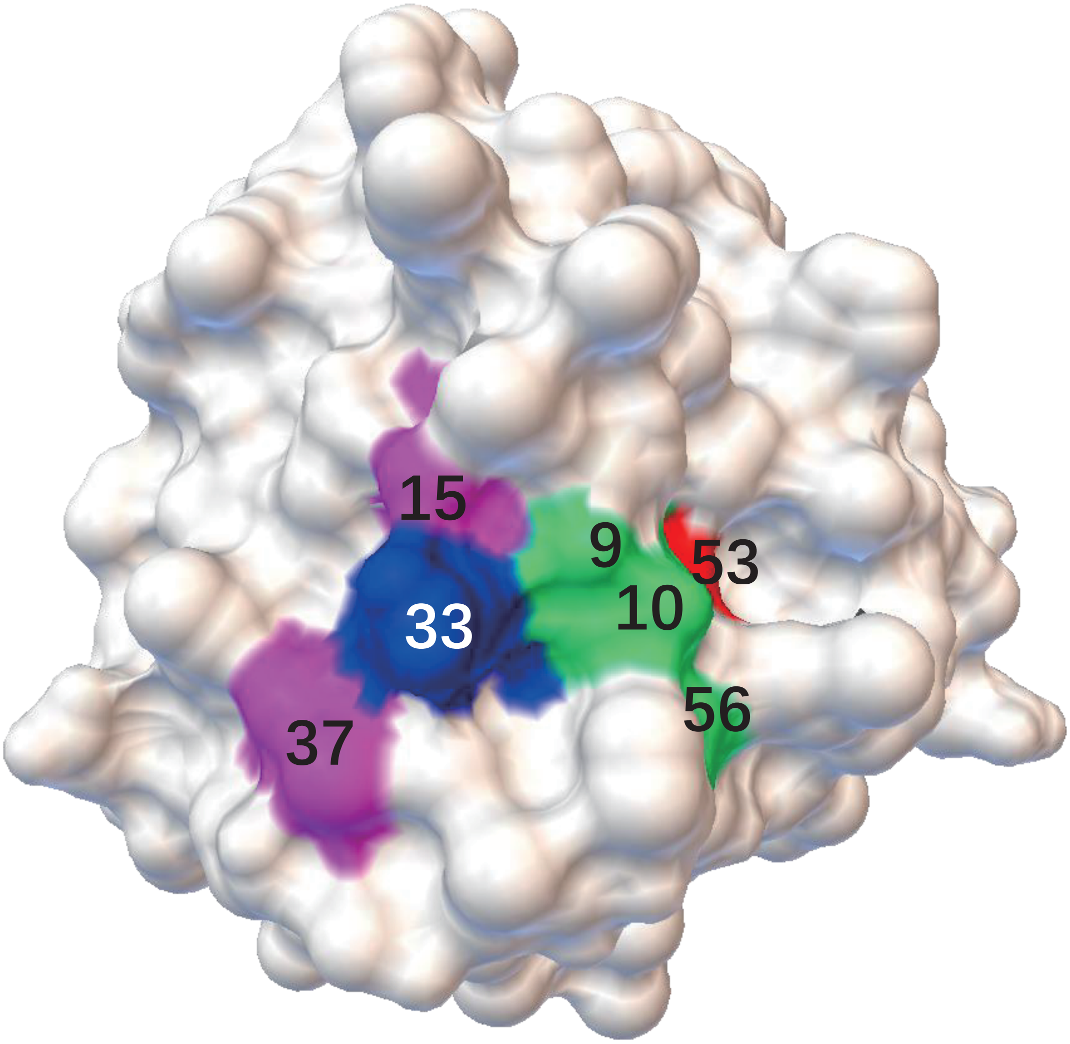

Fig. S8. An example of bridging positions optimized for specific interaction pattern. The position 33 (colored in blue) bridges the positions (9, 10 and 56, green) near the active site 53 (red), and distal positions (15 and 37, purple) with direct contacts.

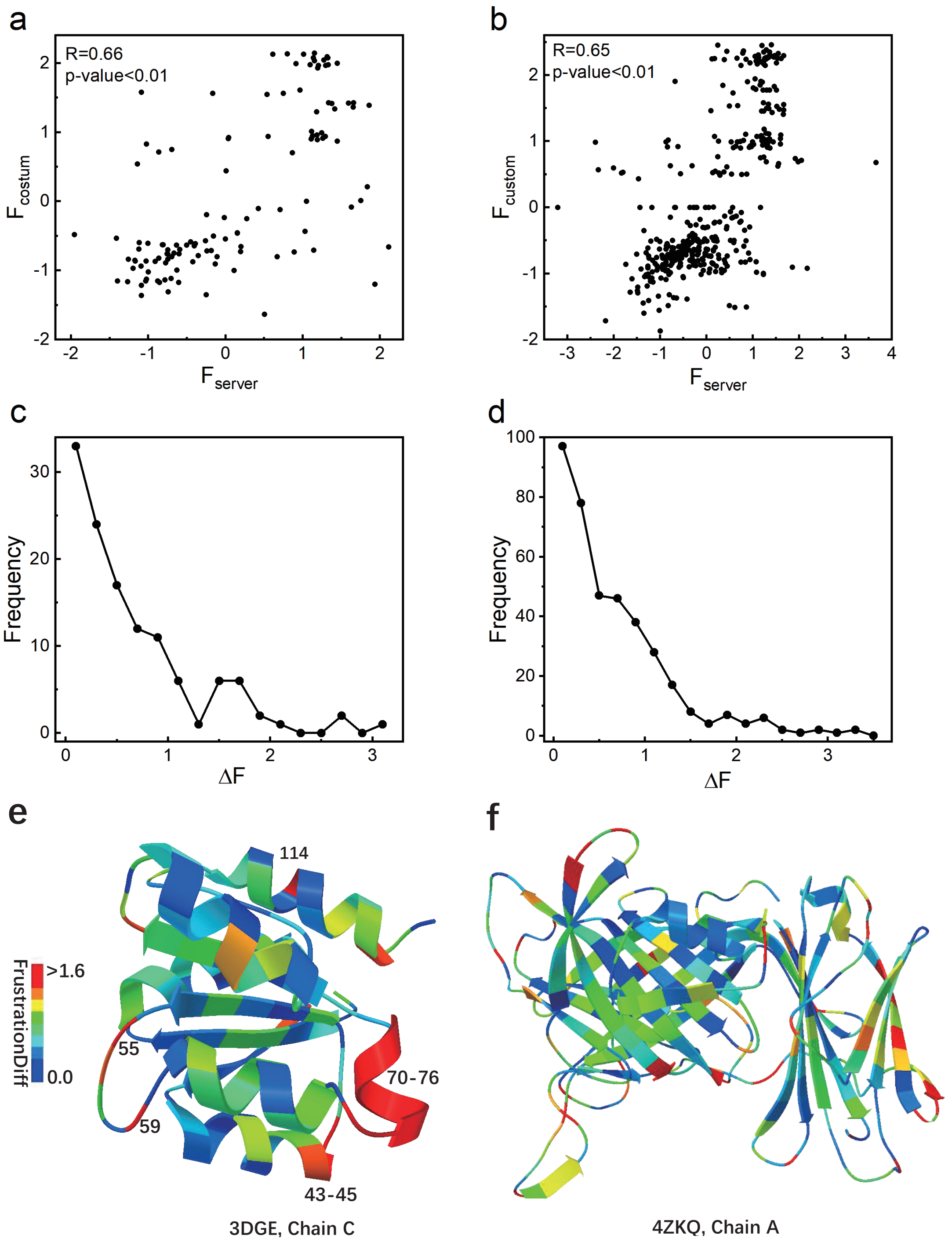

Fig. S9. Comparison of residue-level frustration indexes between Frustratometer server and our customized code. (a) and (b) Scatter plots of residue-level frustration indexes from both versions for 3DGE (chain C, i.e. the Rec domain) and 4ZKQ (chain A). (c) and (d) The frequencies of frustration index difference $\Delta F = |F_{custom} - F_{server}|$ for 3DGE and 4ZKQ. (e) and (f) ΔF mapping on the structure of 3DGE and 4ZKQ, the positions with ΔF>1.6 are all colored red and labeled for 3DGE.

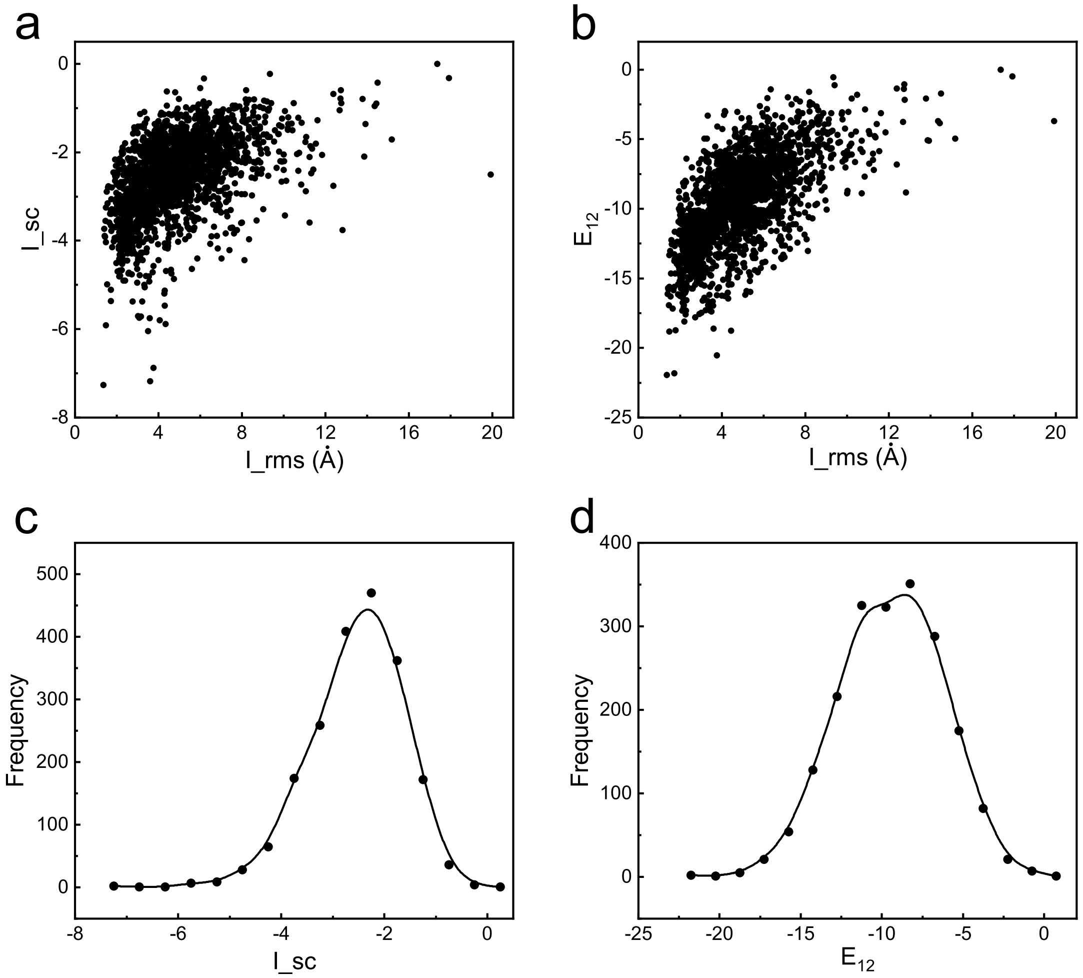

Fig. S10. The conformation ensemble of Rec domain binding with HK. (a) and (b) Energy-conformation relationships of the binding conformation ensemble. (c) and (d) Gaussian-like distribution of binding energies. I_sc means the binding energy scored by Rosetta atomic potentials and E_{12} means the binding energy scored by residue-level MJ matrix, I_rms is the interfacial root-mean-square deviation (RMSD) of interface residues between native binding conformation and binding decoys.

Table S1. Top 16 conserved positions of Rec domain.

Position Index	Position Conservation (C_i)
53	2.989
10	2.957
102	2.914
106	2.790
105	2.742
61	2.569
57	2.531
98	2.468
101	2.432
83	2.290
9	2.173
107	2.127
56	2.111
78	2.054
99	2.046
84	1.985

Table S2. Top-ranked 51 coupling conservations between positions.

Intramolecular Coupling				
**Number**	**Position Index**	**Position Index**	**Coupling Conservation (C_{ij})**	**Distance (Å)**
1	100	115	2.144	9.344
2	94	115	1.939	15.469
3	94	100	1.895	10.666
4	9	56	1.858	7.691
5	55	94	1.809	13.899
6	111	115	1.763	7.636
7	90	100	1.687	16.472
8	55	115	1.662	21.759
9	55	100	1.584	21.511
10	69	100	1.563	12.033
11	95	115	1.557	13.988
12	90	94	1.544	7.242
13	90	115	1.543	18.636
14	69	115	1.497	19.644
15	55	90	1.489	11.285
16	102	115	1.455	9.743
17	114	115	1.420	5.793
18	94	111	1.394	19.082
19	100	111	1.379	15.683
20	102	111	1.351	9.427
21	100	102	1.351	12.14
22	94	102	1.335	7.317
23	65	79	1.325	7.495
24	95	100	1.322	8.408
25	100	114	1.300	14.062
26	107	111	1.296	8.359
27	84	100	1.291	19.266
28	107	115	1.290	11.754
29	84	102	1.259	10.602
30	84	115	1.243	17.399
31	101	115	1.215	6.803
32	52	115	1.212	14.213
33	95	114	1.209	10.131
34	69	94	1.209	18.817
35	56	115	1.204	22.229
36	55	81	1.191	11.906
37	84	94	1.167	13.215
38	82	115	1.162	11.29
39	94	95	1.157	4.533
40	55	102	1.153	14.741
41	55	84	1.144	8.371
42	55	111	1.111	22.025
**Intermolecular Coupling**				
**Number**	**Position Index**	**Position Index**	**Coupling Conservation (C_{ij})**	
43	264	115	1.668	
44	264	100	1.648	
45	264	55	1.409	
46	264	90	1.281	
47	262	115	1.274	
48	264	102	1.272	
49	264	94	1.269	
50	259	55	1.235	
51	264	69	1.101	

Table S3. First-order conservation of 19 positions involved in highly coupling conservations.

Position Index	Position Conservation (C_i)
9	2.173
52	1.542
55	1.640
56	2.111
82	1.522
84	1.985
90	1.479
107	2.127
79	1.829
81	1.699
65	1.355
69	1.860
94	1.630
95	1.455
100	1.807
102	2.914
111	1.608
114	1.203
115	1.734
259	1.477
262	1.691
264	1.902

Table S4. Minimal frustrations required in NSs, FSs and FBSs.

Minimal frustration	Common folding	Common binding	Specific folding	Specific binding
NSs	Yes	Yes	No	No
FSs	Yes	No	Yes	No
FBSs	Yes	Yes	Yes(mostly)	Yes

Table S5. The positions with large change of local frustration ($|\Delta F_i| >= 0.70$) between FSs and FBSs.

Number	Position Index	F_i^{FS}	F_i^{FBS}	ΔF_i
1	14	0.181	1.511	1.330
2	16	-0.572	1.034	1.606
3	20	-0.241	1.279	1.520
4	23	-0.110	0.775	0.885
5	54	0.519	1.330	0.811
6	58	-0.373	0.429	0.802
7	84	-0.602	1.190	1.792
8	85	-0.015	1.208	1.223
9	86	-0.437	1.032	1.469
10	104	-0.244	1.267	1.511
11	107	-0.101	1.346	1.447
12	24	-0.278	-0.988	-0.710
13	33	0.041	-0.708	-0.749
14	62	0.540	-0.186	-0.726
15	87	-0.386	-1.29	-0.904
16	90	-0.029	-0.994	-0.965
17	91	0.177	-0.695	-0.872
18	101	0.340	-0.521	-0.861

Table S6. Bridging positions and their interacting positions in the structure-specific interaction patterns for binding.

Bridging Positions	Positions@Binding Surface	Positions not@Binding Surface
23	20,21,24	19,26,27,28,29
33	9,10,56	15,37
54	53,55,56,83	60,61,62,82
62	54,90	65,66,85,93,97
85	54,55,84,86,87,90	62,104
91	87,88,89,90	94,95,96,102
101		79,80,81,95,115,119
104	86,88,102,107	83,85

Table S7. The average occurring frequencies for 20 residues in native proteins.

Residue	Background Frequency
Cys	0.025
Met	0.023
Phe	0.042
Ile	0.053
Leu	0.089
Val	0.063
Trp	0.013
Tyr	0.033
Ala	0.073
Gly	0.072
Thr	0.056
Ser	0.073
Asn	0.043
Gln	0.040
Asp	0.050
Glu	0.061
His	0.023
Arg	0.052
Lys	0.064
Pro	0.052

Table S8. The classification of hydrophobic and hydrophilic residues.

Hydrophobic Residues	Hydrophilic Residues
Leu	His
Phe	Thr
Ile	Gly
Met	Pro
Val	Arg
Trp	Gln
Cys	Ser
Tyr	Asn
Ala	Asp
	Glu
	Lys

Table S9. Folding conformation ensemble represented by seven families of protein domain.

Pfam name	Pfam accession	Number of structure
ABC_tran	PF00005	480
$Pkinase$	PF00069	312
MFS_1	PF07690	15
BPD_transp_1	PF00528	60
$AMP-binding$	PF00501	57
$p450$	PF00067	33
adh_short	PF00106	259

SI Dataset S1 (DatasetS1.xlsx)

4069 HK-RR pairs of native sequences for MSA

SI Dataset S2 (DatasetS2.xlsx)

PDB IDs of 7 protein families for generating folding conformation ensemble

SI Dataset S3 (DatasetS3.txt)

5000 generated random sequences for DHp domain and Rec domain without background frequencies of amino acids in natural proteins

SI Dataset S4 (DatasetS4.txt)

5000 generated random sequences for DHp domain and Rec domain with background frequencies of amino acids in natural proteins

SI Dataset S5 (DatasetS5.txt)

Residue-level frustration indexes for 3DGE and 4KZQ computed by Frustratometer server and our customized code

Supplementary References

References

1. JD Bryngelson, PG Wolynes, Spin glasses and the statistical mechanics of protein folding. *Proc. Natl. Acad. Sci.* **84**, 7524–7528 (1987).
2. SS Plotkin, J Wang, PG Wolynes, Statistical mechanics of a correlated energy landscape model for protein folding funnels. *The J. chemical physics* **106**, 2932–2948 (1997).
3. J Wang, G Verkhivker, Energy landscape theory, funnels, specificity, and optimal criterion of biomolecular binding. *Phys. review letters* **90**, 188101 (2003).
4. JD Bryngelson, JN Onuchic, ND Socci, PG Wolynes, Funnels, pathways, and the energy landscape of protein folding: a synthesis. *Proteins: Struct. Funct. Bioinforma.* **21**, 167–195 (1995).
5. CL Lee, G Stell, J Wang, First-passage time distribution and non-markovian diffusion dynamics of protein folding. *The J. chemical physics* **118**, 959–968 (2003).
6. J Wang, et al., Topography of funneled landscapes determines the thermodynamics and kinetics of protein folding. *Proc. Natl. Acad. Sci.* **109**, 15763–15768 (2012).
7. Z Yan, J Wang, Superfunneled energy landscape of protein evolution unifies the principles of protein evolution, folding, and design. *Phys. Rev. Lett.* **122**, 018103 (2019).
8. DU Ferreiro, JA Hegler, EA Komives, PG Wolynes, Localizing frustration in native proteins and protein assemblies. *Proc. Natl. Acad. Sci. United States Am.* **104**, 19819–19824 (2007).
9. RG Parra, et al., Protein frustratometer 2: a tool to localize energetic frustration in protein molecules, now with electrostatics. *Nucleic acids research* **44**, W356–W360 (2016).
10. GA Papoian, J Ulander, PG Wolynes, Role of water mediated interactions in protein- protein recognition landscapes. *J. Am. Chem. Soc.* **125**, 9170–9178 (2003).
