# Supplementary material for: Evolution shapes interaction patterns for epistasis and specific protein binding in a two-component signaling system
Source: Commun Chem. 2024 Jan 17;7:13. doi: 10.1038/s42004-024-01098-2 (PMC10794238; doi:10.1038/s42004-024-01098-2)
Supplement: Supplementary file 3 — Description of Additional Supplementary Files [file 42004_2024_1098_MOESM3_ESM.pdf]

Description of Additional Supplementary Files

File name: Supplementary Dataset 1

Description: 4069 HK-RR pairs of native sequences for MSA.

File name: Supplementary Dataset 2

Description: PDB IDs of 7 protein families for generating folding conformation ensemble.

File name: Supplementary Dataset 3

Description: 5000 generated random sequences for DHp domain and Rec domain without background frequencies of amino acids in natural proteins.

File name: Supplementary Dataset 4

Description: 5000 generated random sequences for DHp domain and Rec domain with background frequencies of amino acids in natural proteins.

File name: Supplementary Dataset 5

Description: Residue-level frustration indexes for 3DGE and 4KZQ computed by Frustratometer server and our customized code.
